# Assessment of Malaria Predisposing Factors among Crop Production Farmers Attending the Ndop District Hospital, Northwest Region of Cameroon

**DOI:** 10.1155/2020/1980709

**Published:** 2020-08-01

**Authors:** Nfor Omarine Nlinwe, Tamombi Akonji Emmanuel Ateh

**Affiliations:** Faculty of Health Sciences, Department of Medical Laboratory Science, The University of Bamenda, P.O. Box 39 Bambili, Bamenda, North West Region, Cameroon

## Abstract

The widespread impacts of malaria in the tropical regions of the developing world are not only on healthcare issues but also an agricultural output. Malaria causes manpower loss when it strikes farmers at critical planting, weeding, and harvesting times. Given the above, the expected outcome to malaria prevention programs in farming communities remains a far cry, especially where the predisposing factors are not properly identified and long-lasting solutions proffered. Consequently, this study was designed to assess the malaria predisposing factors among the crop production farmers attending the Ndop District Hospital. The microscopy method was used to determine the presence of malaria parasitaemia. The following categorical variables were considered predisposing factors: Sex, history on malaria illness/treatment, use of long-lasting insecticide nets (LLINs) and knowledge on malaria transmission/prevention. A four-point Likert-type rating scale was adopted for the scoring of the responses given on the predisposing factors, while Fisher's exact test was used to assess the associations between malaria and each of the predisposing factors. The prevalence of malaria parasitaemia among the crop production farmers was 20% (143/715). The predisposing factors tested were found to be significantly associated to the occurrence of malaria among the crop production farmers. Therefore, the combination of improved existing and innovative malaria control strategies may possibly ensure sustained malaria decrease among the farmers in the Ndop Health District.

## 1. Introduction

The most recent world malaria report observed that there were 228 million cases of malaria in 2018 compared to 231 million cases in 2017, resulting to an estimated 405,000 and 416,000 malaria deaths in 2018 and 2017, respectively [1]. The WHO African Region was home to 93% of malaria cases and 94% of malaria deaths in 2018 [1]. A previous study revealed that malaria has the most widespread impact on growth and development in the tropical and equatorial areas of the developing countries [2]. In fact malaria remains prevalent in Cameroon with an estimated annual malaria suspected cases being roughly 3.3-3.7 million in health services [3]. In addition to the cost on health care, malaria cause loss of manpower hours and slows adoption of improved agricultural practices [4]. Thus, it has a significant impact on food security which affects peoples' health. Moreover the agricultural practice in Cameroon is chiefly rain fed and labor intensive, subjecting agricultural output to the state of the farmers' health [5]. Agriculture has therefore been seen as one of the causes of increased intensity of malaria around the world [4, 6–8], due to the fact that it favors the presence of mosquito breeding sites which could also be malaria vectors [4, 9–12].

The fundamental to acquiring success in malaria prevention and control worldwide is access to prompt diagnostic and effective malaria treatment, yet with symptom onset, few fevers are treated with effective antimalarial within 24 hours [13]. The Roll Back Malaria partnership target to ensure 80% of those suffering from malaria have access to appropriate treatment within 24 hours of onset of symptoms by 2010 [14]. But most African countries failed to meet this target [15–18]. However, with the integrated community case management which involved the use of community health workers (CHW), clinical/laboratory diagnosis and treatment of especially target groups with difficult access to health care services were ensured [19–21]. Out of 597 Community Health Workers trained in the Northwest (NW) Region of Cameroon, about 31.99% (191/597) were those trained for the Ndop Health District [22]. In Cameroon, the challenges however associated with this approach are insufficient supervision and motivation of the community health care workers, frequent and long absence of supplies for malaria diagnosis, and treatment [20]. Concerning malaria treatment, the artemisinin-based combination therapies (ACTs) has been evaluated in Cameroon [19, 23–27] with proofs of its continuous efficacy [23, 24]. Treatment with artesunate-amodiaquine (ASAQ) was equally effective in the study area [22]. Nevertheless, it was reported that more than 50% of the population in Cameroon has access to fake and counterfeit drugs which constitutes approximately 50% of the drugs sold in the market or in some private health care providers [19, 28, 29]. In fact in recent years, fake antimalarials have been reported to cause the death of 64,000 to 158,000 people in Africa [30]. Meanwhile, in Tororo Uganda, in spite of the provision of early treatment with ACTs, malaria prevalence seemed to be very high [31]. In fact, studies have indicated the rise in malaria prevalence in Uganda, hence the need for additional malaria control interventions [31].

In addition to effective malaria treatment, the proper use of insecticide-treated mosquito bed nets (ITNs) is a major preventive method used in malaria control [13, 32]. With a long-term assistance from international partners [19, 33], Cameroon has been actively involved in malaria control interventions with the distribution of over 20 million free long-lasting insecticidal nets (LLINs) by 2018 [3]. In fact, these efforts had been fruitful, with about 17% (from 41% to 24.3%) reduction in the national prevalence of malaria cases between 2000 and 2015 and a 54% (from about 13,000 to 6000 per year) reduction in malaria-associated deaths [3]. In the Ndop Health District which has been shown to be dominated by *Anopheles gambiae* s.s., the reduction of indoor transmission has been effective because of the use of LLINs [34]. The good coverage (about 67.3%) of LLINs in Ndop Health District might be responsible for the change in vector behavior, making them more exophilic [35].

However, the use of LLINs would have been more rewarding save for the insufficient collaboration by the Cameroonian population. Despite nationwide sensitization campaigns [36], the performance of LLINs is affected by the variation between ownership and actual usage, at the different epidemiological settings in Cameroon [29, 36–40]. In Cameroon during the 2011 mass distribution of LLINs, a national communication campaign was launched to enhance consistent usage of the LLINs [36]. The national mass media communication was found to strongly improve on the usage of LLINs [36]. Despite this improvement, about 77% of the population owns at least a treated net and only about 58% of them use the nets regularly [3].

However, in Cameroon, there is a rapid extension of insecticide resistance mostly to pyrethroids and dichloro-diphenyl-trichloroethane (DDT) in the main malaria vectors across the nation [41]. In Burkina Faso, care seeking for malaria (among children younger than five years), was not affected by insecticide-treated net campaign, even though there was an increase in the reported use of the bed nets [42]. Also, in spite of the provision of LLINs, malaria prevalence in Tororo, Uganda, seemed to be very high, hence the need for additional malaria control interventions [31]. The improper use of ITNs and resistance to pyrethroid insecticide in Western Kenya were some of the contributing factors to malaria resurgence [43].

Because of an increase in malaria infection rates during rainfalls, this usually accounts for the decrease in agricultural output [44]. The average prevalence of malaria in April, May, and June in the Ndop Health District was approximately 1.44%, 1.86% and 1.36%, respectively, summing up to about 4.66% of malaria cases throughout this important farming season [22]. As malaria strikes farmers in this critical planting, weeding, and harvesting times of April, May, and June, it leads to manpower hours lost [6]. Days lost to malaria illness are assumed to have caused a drop in the percentage of crops harvested, especially among small-scale farmers. In 2017 the estimated output in Ndop area was aggregated to more than 2220 tons, which dropped to less than 2214 tons in 2018 [45]. Therefore this study was designed to assess malaria predisposing factors among crop production farmers attending the Ndop District Hospital.

## 2. Materials and Methods

### 2.1. Study Area and Population

This study was carried out in the Ndop District Hospital located in the Ndop Health District, which is one of the 19 Health Districts in the Northwest (NW) Region. The NW Region is one of the ten regions of Cameroon. The Ndop Health District has 15 Health Areas, with 29 Health facilities. Out of the 29 Health facilities, 16 are public, 7 are private denominational, and 6 are pPrivate secular. Ndop is located at latitude 5.9941° N and longitude 10.4453° E and lies on 1185 m above the sea level, with a tropical climate. The average annual temperature recorded at the time of the study was 21.9°C, with a climate characterized by a long rainy season (March to November) with rainfall averaging between 1800 and 2500 mm per year and average relative humidity of 97% to 98% [46]. The Ndop Health District is located in the malaria “endemic and seasonal” zone of Cameroon, characterized by a long seasonal transmission of 4 to 6 months, with an average of twenty registered infective bites/man/month [19, 47]. *Anopheles gambiae*, *Anopheles arabiensis*, *Anopheles coluzzii*, *A*. *ziemanni*, and *Anopheles funestus* are the major malaria vectors in the study area [19, 47]. In an earlier study, *A*. *ziemanni* was the main malaria outdoor or indoor vector, with the man biting rate for the vectors ranging from 6.75 to 8.29 bites per person per night (b/p/n) [35]. In the Ndop Health District, it was observed that A. ziemanni could sustain the transmission of malaria on its own [35].

The Ndop District hospital acts as the referral hospital for the Ndop Health District. The average (2015 and 2016) annual malaria prevalence was approximately 9.81% and 6.72% in the Ndop Health District and the Northwest (NW) Region, respectively [22]. Meanwhile, the average national malaria prevalence in Cameroon has been reported to vary from 13.08% to 14.64% [3]. Malaria prevalence is typically at its peak in the months of April, May, and June. In the Ndop Health District, in 2016 for example, 14.72%, 18.93%, and 13.86% of the annual malaria cases were recorded in April, May, and June, meanwhile 3.36% and 3.72% were recorded in September and December, respectively [22]. In the North West Region in general, 13.54%, 15.14%, and 12.42% of the annual malaria cases were recorded in April, May, and June, meanwhile 6.18% and 4.81% were recorded in December and February, respectively [22]. Annually, this accounted for about 38% of patient's hospitalization and 3% mortality in the Ndop Health District, whereas in the Northwest (NW) Region in general, it accounted for about 30% of patient's hospitalization and 4% mortality [22].

Ndop Health District area has suitable soils for agriculture and pasture. The level landed nature of the greater part of Ndop has made for the development of swamps and marshes, with water logged soils that support the cultivation of rice. There are approximately 187,348 inhabitants in Ndop Health District and their main economic activity is agriculture: (tea, rice, oil, palm, coffee, maize, beans, Irish potato, yam plantain, banana, Arabica coffee, garden crops, and a variety of fruits). There are also civil servants, some petit traders, and those vested with activities in the transport sector particularly bike riders.

### 2.2. Ethical Consideration

The ethical clearance for this study was gotten from the Ethical Review Committee of the University of Bamenda. The ethical clearance number is 2018/0026/UBa/IRB. Signed informed consents were gotten from those who accepted to be enrolled into the study.

### 2.3. Study Design/Study Participants

This is a cross sectional as well as retrospective study (time series) carried out within 12 months, starting from June 2017 to May 2018. The length of the study period is to enable a proper estimation of the association between the categorical variables (obtained from the past records) and malaria parasitaemia. The inclusion criteria for the study was adult (≥18 years) crop production farmers who consulted at the Ndop district hospital within the study period and were sent to the laboratory for a malaria test. All those who gave their consent to be part of the study by signing the informed consent form were recruited into the study. For the period of one year (12 months), a total of 715 malaria patients visited the laboratory for a malaria diagnostic test.

### 2.4. Laboratory Test Methods

Capillary blood was collected and used to prepare blood films (thick and thin) immediately, following the techniques recommended by Cheesbrough et al. [48]. The prepared blood films were processed and stained with 3% Giemsa staining technique [48]. Two experienced microscopists independently examined duplicate slides. A third experienced microscopist confirmed results with discrepancies. Parasite density per microlitre of blood was estimated following the methods in a previous study [49].

### 2.5. Statistical Analysis

Primary data were obtained from the laboratory analysis and with a structured questionnaire which was designed for the research. The questions contained in the questionnaire elicited data to cover the objectives of the study. A four-point Likert-type rating scale [50] was used for the scoring of the knowledge on malaria transmission/prevention and frequency of the use of insecticide treated mosquito bed nets. The Likert-type rating scale was used because it gives respondents the opportunity to express their opinion and allows them to be neutral if necessary. The points on the Likert-type rating scale for knowledge on malaria transmission/prevention were strongly agree (4), agree (3), partially agree (2), and do not agree (1). Meanwhile, the points on the rating scale for the use of insecticide-treated mosquito bed nets were very frequent (4), frequent (3), averagely frequent, and (2) not frequent (1). The midpoint (a cut-off mean) was gotten by adding 4, 3, 2, and 1, and then dividing the sum by 4 to have 2.5 [50]. Means which were less than 2.5 were interpreted as not knowledgeable on malaria transmission/prevention or no frequent use of LLINs. History of malaria illness and treatment as indicated in their hospital books, in addition to the participant's affirmation, were gotten at the moment of data collection. Those with no account of malaria illness and/or treatment for the past 12 months were categorized under no malaria history and/or treatment. Meanwhile, those with an account of malaria illness and/or treatment for the past 12 months were categorized under positive malaria history and/or treatment. Fisher's exact test which is highly used to assess the associations among categorical variables [51] was used to assess the associations among malaria occurrence and sex, use of LLINs, knowledge on malaria transmission/prevention, and history of malaria infections/treatment in the past 12 months.

## 3. Results

The prevalence of malaria parasitaemia was 20% (143/715). Malaria was detected in all age and sex groups. These infected cases were frequently mild and a majority of those with mild parasitaemia were young adult males. The few severe malaria cases were simply recorded among young adult females. Although the older adult category was the least represented in this study, it had the highest prevalence of malaria. Up to 27.47% (25/91) of the older adults were infected, as compared to 24.34% (92/378) and 10.57% (26/246) of the younger and middle aged adults, respectively. As shown on Table 1, the female farmers constituted up to 58.46% (418/715) of the study participants. Also, the young adults were the most represented age group, constituting 52.87% (715/378) of the study participants. Malaria occurrence among the different sex and age categories were significantly different at *P* value = 0.0001 and < 0.0001, respectively (see Table 1).

All the malaria positive cases had a history of malaria illness and treatment in the past 12 months. None of those who tested negative had been sick of malaria or been on malaria treatment in the past 12 months. Likewise, most of those who tested malaria negative had a history of malaria illness and treatment in the past 12 months. In fact, just about 9.62% (55/572) and 19.23% (110/572) of the malaria negative cases had no history of malaria illness and treatment in the past 12 months, respectively. Generally, most of the study participants were those with history of malaria illness and treatment. Actually, just about 7.69% (55/715) and 15.38% (110/715) of the study participants had no history on malaria illness and treatment in the past 12 months, respectively. The difference in malaria occurrence among those with and without history of malaria illness and treatment was significant at *P* value = 0.0447 and 0.0009, respectively (see Table 2).

Most (51.05%; 73/143) of the study participants who tested malaria positive had knowledge on malaria transmission/prevention but only a few (7.69%; 11/143) of them frequently made use of LLINs in the past 12 months. That means most (92.31%; 132/143) of those who tested malaria positive did not frequently use LLINs in the past 12 months. However among those who tested malaria negative, up to 93.53% and 76.92% had knowledge on malaria transmission/prevention and frequently used LLINs in the past 12 months, respectively. Among those who did not frequently use LLINs, the malaria positive cases were as many as the negative cases. Generally, 85.03% (608/715) and 63.08% (451/715) of the study participants had knowledge on malaria transmission/prevention and frequently used LLINs, respectively. The difference in malaria occurrence among those with and without frequent usage of LLINs and knowledge on malaria transmission/prevention was significant at *P* value < 0.0001 (see Table 3).

## 4. Discussion of Findings

Young adults constituted the highest percentage (52.87%; 378/715) of the study participants. They are dynamic and therefore most suitable for outdoor activities such as farming. The older adult category on the other hand was the least represented (12.73%; 91/715), but with the highest malaria prevalence (27.47%; 25/91). Malaria prevalence among the farmers in general was 20% (143/715). In line with findings from other studies, malaria is usually higher among the older age group. Shwartz et al. reported that individuals older than 40 years were more prone to infections with malaria, compared to those less than 40 years. This was observed in both malaria immune and nonimmune patients, suggesting that increase in age may contribute to higher risk for malaria [52]. This is of particular concern principally among farmers in this study, who are constantly exposed to malaria due to outdoor farming activities at peak mosquito biting periods.

The females who hardly earn income were also more prone to severe parasitaemia. These females fulfill reproductive, home management, and food provision roles for their families, in addition to agricultural activities which cause them to work outdoor [53]. Low economic power among females may determine their health care seeking behavior. If they fall sick, their health situation may get worse if they fail to seek medical attention early. Although malaria was more severe among the females, the males were generally more infected. The prevalence was 10.53% (44/418) and 33.33% (99/297) among the females and males, respectively. In fact, up to 69.23% (99/143) of those who tested positive for malaria were males. Increased vulnerability to malaria by the male farmers could be attributed to longer hours spent at the farms, especially at the peak mosquito biting periods within the day. The females are known to shuttle between domestic and farming activities, usually spending the dusk period of the day performing domestic activities [54]. In line with findings from Herdiana et al., malaria exposure patterns may rise from gender roles and gender dynamics, hence contributing to different vulnerabilities [55, 56]. In nonmalaria endemic areas, malaria disease severity was not significantly associated to gender [57], but gender roles were further proven to determine malaria exposure in malaria endemic areas [55, 56].

The 20% (143/715) malaria prevalence in this study is considered high especially with imminent effects, not only on the farmers' health but also on agricultural output. Such effects are obvious, with malaria prevalence usually at its peak in the critical farming season. Earlier studies reported that malaria has been shown to cause not less than 10 days of incapacitation on susceptible farmers in a month [6]. In the current study, all the malaria positive cases had a history of malaria illness and treatment in the past 12 months, likewise most of those who tested malaria negative. Since there was a significant difference in malaria occurrence between those with and without history of malaria illness in the past 12 months, history of malaria illness/treatment could be considered a predisposing factor. Moreover, malaria resurgence caused by the absence of treatment is a possible risk factor among individuals in endemic areas where reactivation and reinfection of the disease could coincide. However, an adult with history of previous clinical malaria in a malaria nonendemic country has significantly lower risks of malaria illness [58]. However, this was reported in a malaria nonendemic zone. Farming activities in a malaria holoendemic area like the Ndop Health District could enhance malaria persistence through reinfection, even after previous treatment.

Although the Ndop Health District had benefited from the integrated community case management with 191 trained community health care workers [22], accessing prompt treatment remains a challenge to some of the farmers. Among the study participants, 92.31% (660/715) had a positive malaria history while 15.38% (110/715) had not been on any prescribed malaria treatment in the past 12 months (see Table 2). Therefore, there were some farmers who had untreated malaria history. Moreover, all those who tested malaria positive had a history of malaria treatment in the past 12 months, suggesting possible treatment failure and/or reinfection in some cases. The pending challenges of fake and counterfeit drugs could contribute to malaria treatment failure or resurgence [19, 28, 29]. Similarly, in other parts of Africa, malaria prevalence remained high despite provision of early treatment with ACT [31]. Therefore, in resource constrained health systems such as Cameroon, ensuring prompt access to effective malaria treatment remains a challenge [13]. Since the effects of fake drugs cannot be underestimated, elimination of these fake drugs is absolutely necessary in the effective fight against malaria [19]. Malaria cases should also be tracked by proper detection and treatment.

The practice of LLIN usage was poor among those who tested malaria positive. In fact, up to 92.31% (132/143) of them did not frequently use LLINs in the past 12 months (see Table 3). Malaria preventive measures such as the proper usage of LLINs are an effective measure employed over time to combat malaria transmission in endemic regions [43, 59, 60]. Among those who tested malaria negative in the current study, 76.92% (440/572) had made frequently used LLINs in the past 12 months, thanks to the 67.3% good coverage of LLINs in the Ndop Health District [35]. There were however some few (7.69%; 11/143) malaria-positive cases who frequently used LLINs. Generally, up to 36.92% (364/715) of the farmers did not frequently use the LLINs, despite its good coverage. However, the use of LLINs in this study was 63.08% (451/715). Therefore, in line with findings from other studies, despite ownership of LLINs by some of the study participants, improper and/or lack of usage may be another issue to combat [31, 42, 43].

There is also indication of rapid expansion of DDT, pyrethroid, and carbamate resistance in *A*. *gambiae*, *A*. *coluzzii*, *A*. *arabiensis*, and *A*. *funestus* threatening the performance of LLINs in the Ndop Health District [19]. *A*. *funestus* was also found to be the chief malaria vector in high agricultural activity areas [61]. Although *Anopheles funestus* and *Anopheles arabiensis* were originally indoor vectors, they are however increasingly biting outdoors in recent times, hence contributing more to residual malaria transmission [34]. In an agricultural community like the Ndop Health District, residual transmission is caused by changes in mosquito host seeking behavior and augmented increase in outdoor biting [62]. Hence, emphasizing control of outdoor transmission will enhance efforts to eliminate malaria transmission [62].

A recent study in Yaounde Cameroon reported that A. gambiae s.l. and A. funestus s.l. were the main malaria vectors [3]. The average biting rate of Anopheline was significantly high outdoor than indoor, with seasonal variation in mosquito abundance and biting rate. [3]. This study suggested immediate actions to increase the control of high malaria transmission occurring in Yaoundé and call for immediate actions to improve control policies [3]. These improve control policies could also be implemented nationally. Therefore, in order to significantly reduce malaria burden among the farmers in Ndop Health District, entomological heterogeneity must be considered when designing vector control strategies.

Among the farmers in Ndop Health District, there is very low income due to high cost of the labor intensive activities, with unattractively low producer price [63]. Consequently, their homes and temporary farm houses are poorly designed and highly exposed to malaria transmission. Transfluthrin-treated eave ribbons, a technology which has been shown to possibly complement ITNs, rendered protection to periodic migratory farmers against both indoor and outdoor-biting mosquitoes in rural Tanzania [64]. This technology can also be adapted in the Ndop Health District to control both outdoor and indoor malaria transmission, especially during farmer's periodic stay in temporary huts. This and other new strategies should be developed to alleviate the influence of outdoor and residual transmission in especially agricultural communities [19].

Most (93.53%; 535/572) of the farmers who tested malaria negative had knowledge on malaria transmission/prevention and a few of them (6.47%; 37/572) were not knowledgeable. But 48.95% (70/143) of the farmers who tested malaria positive did not have knowledge on malaria transmission/prevention. In line with previous findings, malaria control strategies will produce little benefits in the absence of adequate knowledge on its transmission/prevention [65, 66]. Although the use of LLINs was shown to reduce indoor transmission in the Ndop Health District [34], the farmers' limited knowledge on outdoor transmission could have contributed to the current malaria prevalence rate of 20%. As a follow-up on the recent third mass distribution campaign of LLINs launched in Cameroon in February 2019, a study was conducted to investigate the impact of door-to-door hang-up and behavior change communication (BCC) campaign, on the use of the bed nets in a rural population [67]. The use of bed nets increased from 75% before the campaign to 92% after the campaign [67].

## 5. Conclusion

There is absolute necessity to integrate control approaches in order to sustain malaria control among the farmers in the Ndop Health District. The already existing policies on malaria control interventions such as the integrated community case management and mass distribution of LLINs should be restructured with inclusions on how to possibly overcome pertinent challenges. The LLIN hang up and BCC campaign should be incorporated alongside the recently launched third mass distribution of LLINs campaign, in order to ensure increased usage of the bed nets. Finally, strict epidemiological and entomological investigative activities are encouraged, so that successful implementation of control interventions can be monitored.

## Figures and Tables

**Table 1:** Distribution of absence/presence of malaria parasitaemia according to sex and age of the farmers.

	Negative (%)	Mild parasitaemia (%)	Moderate parasitaemia (%)	Severe parasitaemia (%)	Row total (%)	Fisher's exact test (*P* value)
Sex						
Males	198 (34.62)	77 (87.5)	22 (66.67)	0	297 (41.54)	=0.0001
Females	374 (65.38)	11 (12.5)	11 (33.33)	22 (100)	418 (58.46)	
Age						
Young adults: ≥18 yrs to ≤35 yrs	286 (50)	48 (54.55)	22 (66.67)	22 (100)	378 (52.87)	
Middle aged adults: >35 yrs to ≤55 yrs	220 (38.46)	26 (29.55)	0	0	246 (34.41)	<0.0001
Older adults: >55 yrs	66 (11.54)	14 (15.91)	11 (33.33)	0	91 (12.73)	
Column total (%)	572 (80)	88 (12.31)	33 (4.62)	22 (3.08)	715	

**Table 2:** Occurrence of malaria according to history on malaria illness and intake of prescribed treatment in the past 12 months.

	Malaria positive (%)	Malaria negative (%)	Row total (%)	Fisher's exact test (*P* value)
History on malaria illness (12 months)				
Positive malaria	143 (100)	517 (90.38)	660 (92.31)	0.0447
No malaria	0	55 (9.62)	55 (7.69)	
History on malaria treatment (12 months)				
Malaria treatment	143 (100)	462 (80.77)	605 (84.62)	0.0009
No malaria treatment	0	110 (19.23)	110 (15.38)	
Column Total (%)	143 (20)	572 (80)	715	

**Table 3:** Occurrence of malaria according to frequent use of LLINs and knowledge on malaria transmission/prevention.

	Malaria positive (%)	Malaria negative (%)	Row total (%)	Fisher's exact test (*P* value)
History on frequent use of ITNs (12 months)				
Frequent use	11 (7.69)	440 (76.92)	451 (63.08)	< 0.0001
No frequent use	132 (92.31)	132 (23.08)	264 (36.92)	
Knowledge on malaria transmission/prevention				
Knowledgeable	73 (51.05)	535 (93.53)	608 (85.03)	< 0.0001
Not knowledgeable	70 (48.95)	37 (6.47)	107 (14.97)	
Column total (%)	143 (20)	572 (80)	715	

## Data Availability

All the data for this study can be acquired from the authors upon request.
